# Supplementary material for: Splice-Junction-Based Mapping of Alternative Isoforms in the Human Proteome
Source: Cell Rep. Author manuscript; Available in PMC 2020 Jan 15. (PMC6961840; doi:10.1016/j.celrep.2019.11.026)
Supplement: 3 [file NIHMS1546469-supplement-3.zip › DF2/PXD0010154/Spleen-202-Q9Y2Q0-AYGQNSQFGDEK.pdf]

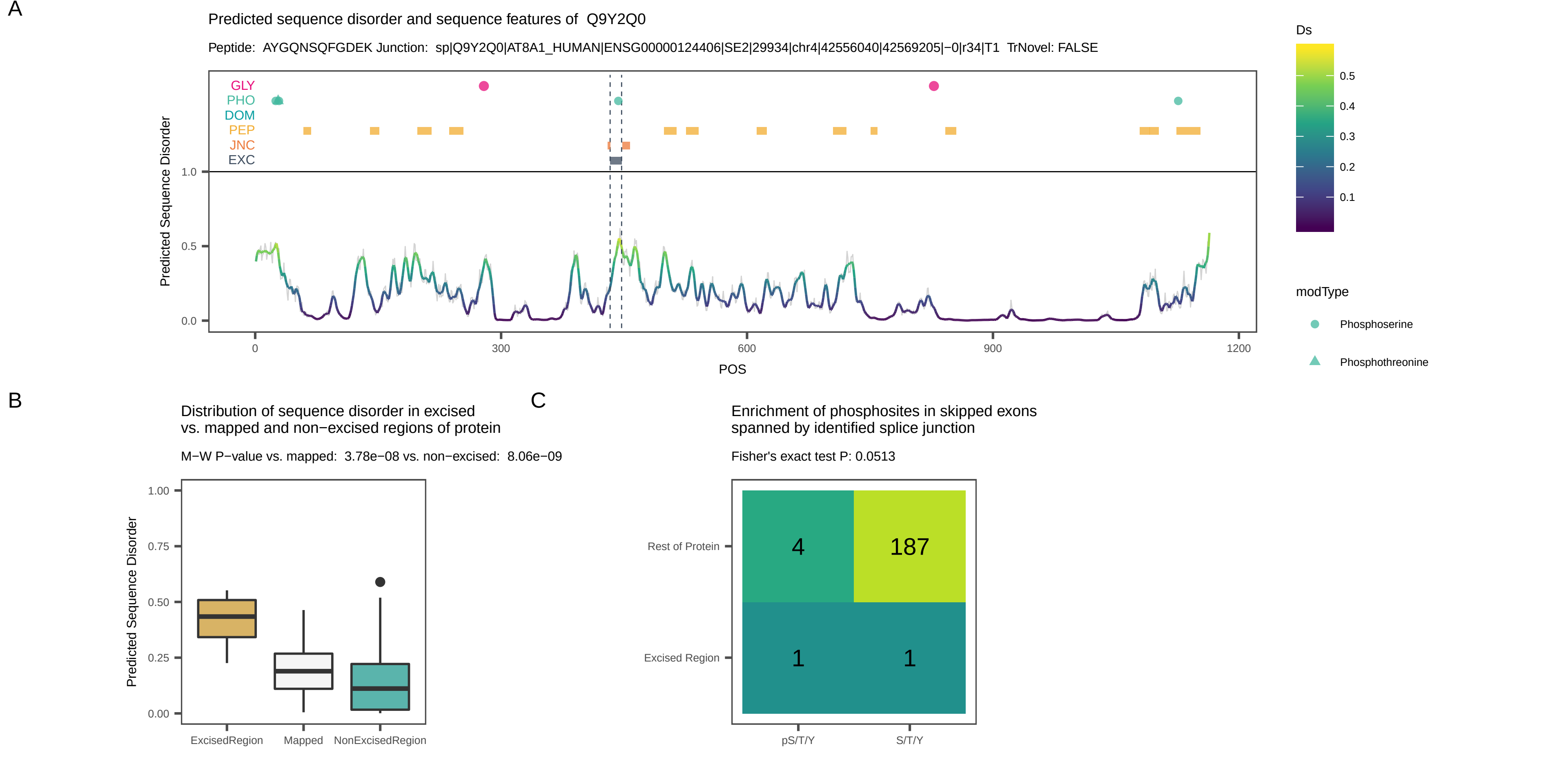
A
Predicted sequence disorder and sequence features of Q9Y2Q0
Peptide: AYGQNSQFGDEK Junction: sp|Q9Y2Q0|AT8A1_HUMAN|ENSG00000124406|SE2|29934|chr4|42556040|42569205|−0|r34|T1 TrNovel: FALSE
GLY
PHO
DOM
PEP
JNC
EXC
Predicted Sequence Disorder
1.0
0.5
0.0
0
300
600
900
1200
POS
Ds
0.5
0.4
0.3
0.2
0.1
modType
Phosphoserine
Phosphothreonine
B
Distribution of sequence disorder in excised
vs. mapped and non−excised regions of protein
M−W P−value vs. mapped: 3.78e−08 vs. non−excised: 8.06e−09
Predicted Sequence Disorder
1.00
0.75
0.50
0.25
0.00
ExcisedRegion
Mapped
NonExcisedRegion
C
Enrichment of phosphosites in skipped exons
spanned by identified splice junction
Fisher's exact test P: 0.0513
Rest of Protein
Excised Region
4
187
1
1
pS/T/Y
S/T/Y
